# Computational Analysis of Single Nucleotide Polymorphisms in Human HIC1 Gene

**DOI:** 10.7759/cureus.56664

**Published:** 2024-03-21

**Authors:** Arora Annanya, Boopathi Priyadharshini, Vasugi Suresh, Elangovan Dilipan

**Affiliations:** 1 Physiology, Saveetha Dental College and Hospitals, Saveetha Institute of Medical and Technical Sciences (SIMATS), Chennai, IND; 2 Physiology, Saveetha Dental College and Hospitals, Saveetha Institute of Medical and Technical Sciences, Chennai, IND; 3 Medical Physiology, Saveetha Dental College and Hospital, Saveetha Institute of Medical and Technical Sciences, Chennai, IND

**Keywords:** wnt signaling pathway, cancer protein, hic1 gene, single nucleotide polymorphism, in silico analysis

## Abstract

Background

A putative tumor suppressor gene called HIC1 (hypermethylated in cancer) is situated at 17p13.3, a locus where the allelic loss occurs often in human malignancies, including breast cancer. Hypermethylated in cancer 1 protein is a protein that in humans is encoded by the *HIC1* gene and it’s a *Homo sapiens *(Human). This gene functions as a growth regulatory and tumor repressor gene. The molecular function of *HIC1* gene includes DNA-binding transcription factor activity, sequence-specific DNA binding, DNA binding, histone deacetylase binding, protein binding, metal ion binding, nucleic acid binding, DNA-binding transcription repressor activity, RNA polymerase II-specific, DNA-binding transcription factor activity, RNA polymerase II-specific. The biological process of *HIC1* gene includes multicellular organism development, negative regulation of Wnt signaling pathway, positive regulation of DNA damage response, signal transduction by p53 class mediator regulation of transcription, DNA-templated, negative regulation of transcription by RNA polymerase II, Wnt signaling pathway, transcription, DNA-templated, intrinsic apoptotic signaling pathway in response to DNA damage, cellular response to DNA damage stimulus. The study aimed to predict the stability and structure of the protein that will arise from single nucleotide polymorphisms (SNPs) in the human HIC1 gene.

Methodology

To investigate the possible negative effects associated with these SNPs, bioinformatic analysis is typically essential. The following tools were employed for forecasting harmful SNPs: scale-invariant feature transform (SIFT), Protein Analysis Through Evolutionary Relationships (PANTHER), nonsynonymous SNP by Protein Variation Effect Analyzer (PROVEAN), and nonsynonymous SNP by Single Nucleotide Polymorphism Annotation Platform (SNAP).

Results

The present study identified a total of 36 SNPs using the SIFT approach, which were shown to have functional significance. Twenty-six were determined to be tolerable, whereas 10 were shown to be detrimental. Out of 20 SNPs, seven (P370A, P646S, R654P, A476T, S400S, D666N, D7V) SNPs were predicted as “Possibly damaging” and seven (L9F, G468R, G490R, L482R, S12W, G489D, S12P) were identified as “probably benign”, and six (R725G, G620S, A56V, E463D, D394N, L338V) were identified as “probably damaging” according to the predictions made by PANTHER tools. The majority of the pixels on the strip were red, indicating that the gene changes may have dangerous consequences. These results highlight the need for more research to fully comprehend how these mutations affect the hic1 protein's function, which is essential for the emergence of different types of cancer.

Conclusion

The current research has provided us with essential information about how SNPs might be used as a diagnostic marker for cancer, given that SNPs may be candidates for cellular changes caused by mutations linked to cancer.

## Introduction

The correlation of HIC1 (hypermethylated in cancer), a candidate tumor suppressor gene, with a CpG-rich region at 17p13.3-also known as the “CpG island” that is aberrantly hypermethylated and transcriptionally inactivated in several common types of human cancer, including breast cancer cell lines, led to its identification recently [1]. In comparison to the genome as a whole, these islands are brief, widely distributed DNA segments that have a high frequency of CpG dinucleotides. About 2% of the sample is linked to the 5′-ends, and thus the transcription starts sites and promoters of all housekeeping genes as well as some tissue-specific genes, have been hypothesized to be locations of interaction between transcription factors and promoters. The predominant location of DNA methylation, though not the only one, is the CpG dinucleotide [2]. The methylation status of the base cytosine influences the overall genomic pattern of chromatin architecture and gene expression [3]. However, more and more research studies are showing that many human diseases, including cancer, are also largely caused by epigenetic alterations, which are identified by DNA methylation and modifications to the histone tail that transmit heritable patterns of gene expression [4]. HIC-1, in addition to the lissencephaly-1 gene (LIS-1), is implicated in the development of Miller-Dieker syndrome (MDS) and is found within the key 350 kb region lost in the majority of cases, according to recent studies [5]. The hypermethylation of HIC-1 leads to transcriptional suppression in a variety of human malignancies, such as hepatocellular carcinomas, medulloblastomas, and astrocytic gliomas.

In the current work, we looked at HIC1's potential function in this tumor type [6]. HIC1's downstream target genes, which include SIRT1, ATOH1, TCF4, CXCR7, CyclinD1, P57KIP2, ephrin-A1, Eph A2, SOX9, and FGF-BP1, have recently been identified as being involved in developmental, proliferation, migration, invasion, angiogenesis, and cell-cycle control [7]. HIC1 encodes a nuclear BTB/POZ protein characteristic with five Kruppel-like C2H2 zinc fingers in the C-terminal region and a BTB/POZ domain at the N-terminus [8]. Positional cloning of HIC1 was made possible in several malignancies by DNA hypermethylation alterations of the NotI restriction sites at the D17S5 locus in 17p13.3 [9]. The most prevalent type of genetic variation in humans is represented by single nucleotide polymorphisms (SNPs), which can be used as a tool for mapping intricate genetic traits1. High-throughput sequencing initiatives generate vast amounts of data, which represent a rich and largely unexplored supply of SNPs [10]. A large majority of these DNA variants are SNPs, which are genomic sites at which there are two different nucleotide residues (alleles) that are both present in a significant portion of the human population [11]. This is especially true for the study of the human genome, where more than a million SNPs, the most prevalent kind of sequence variations across alleles, have been recently identified [12]. SNP discovery is currently of significant interest because a rich catalog of SNPs is anticipated to make large-scale investigations in population genetics and evolutionary biology (3), association genetics (1), functional and pharmaco-genomics (2), positional cloning and physical mapping (4), and population genetics and evolutionary biology easier [13].

The nonsynonymous SNPs (nsSNPs) among them alter the residues of amino acids. These most likely have a significant role in the functional diversity of the encoded proteins in the population of humans [14]. To forecast the potential effects of SNPs on the structure and functionality of Mannose-binding lectin (MBL), several bioinformatics tools were used to evaluate SNP data that were taken from the dbSNP database [15]. In cases of non-familial breast/ovarian cancer, three SNPs Lys312Asn, Cys557Ser, and Asn295Ser have been linked to BRCA1 and BRCA2 mutations [16]. SNPs and small deletions in key *FMR1 *gene domains may result in fragile X syndrome (FXS)-like characteristics [17]. Previously, the in-silico technologies were used to evaluate variations of the *TAGAP *gene [18]. Over 90% of all variations in human nucleic acid sequences can be attributed to SNPs [19]. We looked through the Cancer Genome Atlas (TCGA) database and current publications [20]. The purpose of this investigation was to ascertain the effects of a genetic variant (rs2071676) in the *H1C1 *gene [21]. In-silico research offers strong proof of the different genes.

## Materials and methods

Retrieval of variant datasets

Variant datasets for the *HIC1 *gene of 430 SNPs were retrieved in a variety of methods, from clinical databases to private databases to public databases. Human *HIC1 *gene data were collected from Online Mendelian Inheritance in Man and Ensemble, a public database of bibliographic information about human genes and genetic disorders. SNP data was collected from the National Center for Biotechnology Information (NCBI) dbSNP (SNP database). Protein sequence from the Kyoto Encyclopedia of Genes and Genomes (KEGG).

Deleterious SNP prediction by SIFT

Based on reports, the scale-invariant feature transform (SIFT) can differentiate between amino acid alterations that are detrimental and functionally neutral in mutagenesis studies and human polymorphisms. During this process, relevant protein sequences are found by searching, closely related sequences with comparable functions are chosen, multiple alignments are obtained, and normalized probability for all potential substitutions at each site is established [22]. Calculated probability over 0.05 is predicted to be tolerated, whereas those below 0.05 indicated detrimental intolerant substitutions at each site.

Evolutionary relationships by PANTHER

Based on a mathematical framework for pattern recognition called the Hidden Markov Model (HMM), the PANTHER (Protein analysis through evolutionary relationships) statistical model was developed. HMMs are widely used in bioinformatics for protein structure prediction, sequence alignment, and gene prediction [23].

Prediction of the functional effect of SNAP-2

The purpose of Screening of Non-Acceptable Polymorphism 2 (SNAP2) is to identify functional effects resulting from mutations. In order to classify novel mutations as “acceptable” or “non-acceptable” [23,24].

## Results

Retrieval of variant datasets

Polymorphisms that have been validated or not are included in the dbSNP database. Despite this drawback, we chose to utilize the dbSNP because of its extensive database of allelic frequencies for the majority of HIC1 nsSNPs. It is regarded as the most complete SNP database available. A total of 36 SNPs were isolated, 10 were found to be deleterious and only 26 were tolerated.

Deleterious SNP prediction by SIFT

If nsSNPs are harmful or tolerated, it can be identified using the SIFT approach. Finding probable disease candidates from missense changes is made possible in large part by the SIFT technology. As opposed to those with a probability of ≥0.05, which were predicted to be tolerated, amino acid changes with a normalized probability cutoff value of ≥0.05 were predicted to be harmful. SIFT tools were used to predict and identify the deleterious and tolerated SNPs. Out of 36 SNPs, 10 (R725G, R706G, G620S, G601S, R654P, R635P, A56V, A37V, D7V, S12W) SNPs were predicted as “harmful” and 26 (P351A, P370A, P646S, P627S, A457T, A476T, L9F, G468R, G449R, G471R, G490R, S400S, S381S, E463D, E444D, L482R, L463R, D394N, D375N, D647N, D666N, L338V, L319V, G470D, G489D, S12P) were tolerated by SIFT tools. Hence, those 10 SNPs were high-risk SNPs (Table 1).

**Table 1:** List of nsSNPs found to be functionally significant by SIFT tool. SIFT: scale-invariant feature transform; nsSNPs: nonsynonymous single nucleotide polymorphisms

rsID	Amino acid change	SIFT Prediction	Score
rs1063317	R725G	DELETERIOUS	0.013
rs1063317	R706G	DELETERIOUS	0.014
rs182733310	P351A	TOLERATED	0.831
rs182733310	P370A	TOLERATED	0.936
rs188260013	P646S	TOLERATED	0.295
rs188260013	P627S	TOLERATED	0.325
rs199947545	G620S	DELETERIOUS	0.029
rs199947545	G601S	DELETERIOUS	0.029
rs200496232	R654P	DELETERIOUS	0.008
rs200496232	R635P	DELETERIOUS	0.009
rs200866682	A56V	DELETERIOUS	0.004
rs200866682	A37V	DELETERIOUS	0.004
rs201599490	A457T	TOLERATED	0.408
rs201599490	A476T	TOLERATED	0.468
rs201780325	L9F	TOLERATED	0.656
rs202115526	G468R	TOLERATED	0.519
rs202115526	G449R	TOLERATED	0.522
rs370020453	G471R	TOLERATED	0.051
rs370020453	G490R	TOLERATED	0.066
rs370995439	S400S	TOLERATED	1
rs370995439	S381S	TOLERATED	1
rs371242587	E463D	TOLERATED	0.091
rs371242587	E444D	TOLERATED	0.104
rs371934522	L463R	TOLERATED	0.285
rs371934522	L482R	TOLERATED	0.303
rs373366887	D394N	TOLERATED	0.323
rs373366887	D375N	TOLERATED	0.324
rs373603534	D647N	TOLERATED	0.442
rs373603534	D666N	TOLERATED	0.446
rs373870177	L338V	TOLERATED	0.358
rs373870177	L319V	TOLERATED	0.51
rs375835534	D7V	DELETERIOUS	0
rs376178946	S12W	DELETERIOUS	0.011
rs376477815	G470D	TOLERATED	0.06
rs376477815	G489D	TOLERATED	0.104
rs377629449	S12P	TOLERATED	0.282

Evolutionary relationships by PANTHER

PANTHER tools were used for gene prediction, sequence alignment, and protein structure prediction. Out of 20 SNPs, seven (P370A, P646S, R654P, A476T, S400S, D666N, D7V) SNPs were predicted as “Possibly damaging” and seven (L9F, G468R, G490R, L482R, S12W, G489D, S12P) were identified as “probably benign”, and six (R725G, G620S, A56V, E463D, D394N, L338V) were identified as “probably damaging” by PANTHER tools. Hence, those seven SNPs were high-risk SNPs (Table 2).

**Table 2:** List of SNPs predicted to be damaging related by PANTHER. SNPs: single nucleotide polymorphisms; PANTHER: Protein Analysis Through Evolutionary Relationships

Substitution	Preservation Time	Message	Pdel
R725G	455	Probably damaging	0.57
P370A	361	Possibly damaging	0.5
P646S	361	Possibly damaging	0.5
G620S	456	Probably damaging	0.57
R654P	361	Possibly damaging	0.5
A56V	456	Probably damaging	0.57
A476T	361	Possibly damaging	0.5
L9F	97	Probably benign	0.19
G468R	98	Probably benign	0.19
G490R	98	Probably benign	0.19
S400S	361	Possibly damaging	0.5
E463D	456	Probably damaging	0.57
L482R	98	Probably benign	0.19
D394N	455	Probably damaging	0.57
D666N	361	Possibly damaging	0.5
L338V	455	Probably damaging	0.57
D7V	361	Possibly damaging	0.5
S12W	97	Probably benign	0.19
G489D	97	Probably benign	0.19
S12P	97	Probably benign	0.19

Prediction of the functional effect of nonsynonymous SNP by SNAP

**Figure 1:**
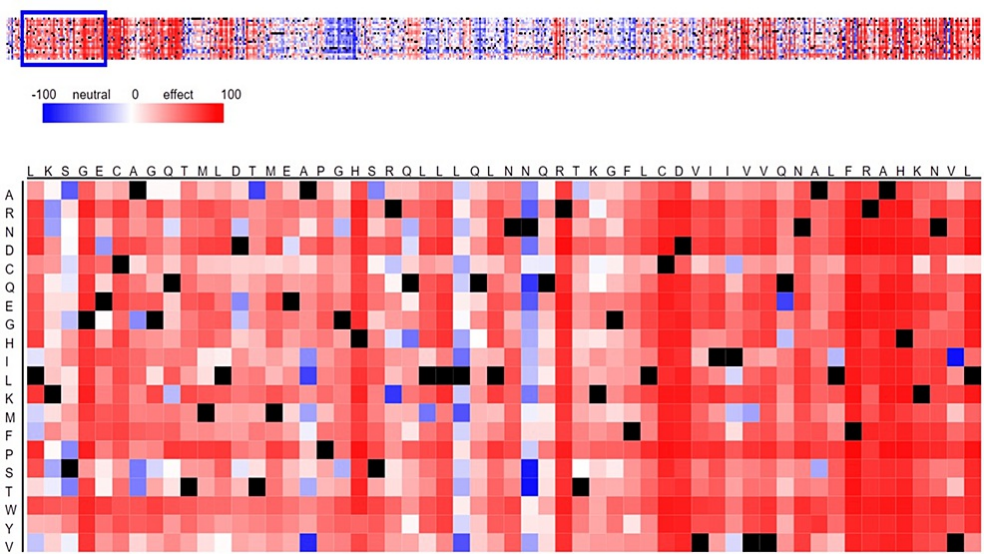
Predicting functional effects of sequence variants of HIC1 gene by SNAP2 SNAP2: screening of non-acceptable polymorphism 2

SNAP2 often offers a level of confidence, which may be shown by the intensity of the colors. The current findings demonstrate that the gene had a neutral impact, as shown by the presence of blue cells, while its negative effect was indicated by the presence of red dots. With the exception of one patch that has more blue pixels than the rest, the strip is primarily covered in red pixels (Figure 1), indicating that it is potentially hazardous. These hypotheses may aid in the design of additional studies to determine the impact of these mutations on the function of the hic1 protein, which is critical to the development of numerous cancer types.

## Discussion

HIC1 is a tumor suppressor gene that aids in the prevention of cancer. In-silico analysis is being utilized to find these mutations and comprehend how they contribute to the emergence of cancer. The potential effects of these SNPs on gene function and human health can be foreseen using in-silico analysis of SNPs in the human *HIC1 *gene with a range of methods. A sort of genetic variation found in the human genome called SNPs can cause substitutions in amino acids, which can change the structure and function of proteins. Amino acid substitution is the outcome of nsSNPs, which have been linked to a variety of hereditary illnesses. In-silico methods, on the other hand, screen for potentially harmful variations more quickly and affordably by predicting the functional impact of nsSNPs through computational methods [25]. The human HIC1 gene's SNPs were gathered from the NCBI dbSNP database. Utilizing SIFT, Protein Variation Effect Analyzer (PROVEAN), and PANTHER tools to retrieve and analyze variant databases specifically, SNPs has yielded important insights into the possible functional implications of genetic variations. HIC1 belongs to the POK/ZBTB protein family, which also contains a number of TFs.

Numerous studies have been conducted on HIC1 in relation to cancer. It functions as a tumor suppressor and is connected to a number of important carcinogenesis processes, such as cell migration and proliferation [26]. To comprehend genetic variants and their possible effects on protein function, SNPs must be identified and characterized. In order to forecast the harmful and tolerated SNPs for this work, we collected a dataset of (36) SNPs from the dbSNP database and used computational methods, namely SIFT, PANTHER, PROVEAN tools. A survey of research supports our projected changes for a few examined SNPs, such as rs16965628, rs140700, and rs25532. While rs6295 and rs6311 were identified as potentially dangerous and relationships were discovered, rs6352, rs6355, rs1923882, and rs2066713 were identified by all prediction methods as not potentially dangerous and no associations with phenotypes were discovered [27]. Using SIFT tools enabled the prediction of deleterious and tolerated SNPs within the database. The majority of SNPs (10 out of 36) (R725G, R706G, G620S, G601S, R654P, R635P, A56V, A37V, D7V, and S12W) were classified as “harmful” by SIFT, indicating a high risk associated with these genetic variations. These SNPs may cause variations to the amino acid sequence, which may have a negative impact on the structure or function of the protein. On the other hand, SIFT categorized 26 SNPs as tolerated, they are P351A, P370A, P646S, P627S, A457T, A476T, L9F, G468R, G449R, G471R, G490R, S400S, S381S, E463D, E444D, L482R, L463R, D394N, D375N, D647N, D666N, L338V, L319V, G470D, G489D, S12P. It is less expected that these mutations will seriously damage protein function. All the SNPs had been sent for further analysis. Protein structure prediction, sequence alignment, and gene prediction were all done using PANTHER techniques, PANTHER tools identified seven SNPs (P370A, P646S, R654P, A476T, S400S, D666N, and D7V) as “Possibly damaging,” seven (L9F, G468R, G490R, L482R, S12W, G489D, and S12P) as “probably benign,” and six (R725G, G620S, A56V, E463D, D394N, and L338V) as “probably damaging” out of 20 SNPs. These seven SNPs were therefore high-risk SNPs. Choosing the best web-based bioinformatics application to utilize takes a lot of time and effort because there are so many available. In order to assist researchers in quickly analyzing and choosing the most promising SNPs for drug discovery, we present an overview of cutting-edge bioinformatics tools here [28].

The degree of confidence that SNAP2 frequently provides can be seen in the colors’ intensity. According to the current findings, the gene’s positive effect was represented by red dots, and its negative effect was represented by blue cells. The strip was mainly covered in red pixels, suggesting that it may be dangerous, with the exception of one patch that contains more blue pixels than the rest (Figure 1). These theories could help shape future research to find out how these mutations affect the HIC1 protein's function, which is essential to the growth of many cancer forms. Genetic variations can affect an individual's susceptibility to disease as well as the therapeutic response and side effects caused by drugs. Researching the impacts of functional exon SNPs in disease-correlated proteins can aid in the development of novel medications that counteract the effects of these mutations in the general population. The current work used a variety of in-silico techniques to estimate the effects of nsSNPs of TUFT1 [29]. The challenge of precisely forecasting the context-dependent effects of SNPs is one of the drawbacks of in-silico analysis. Depending on the kind of cells, stage of growth, and external variables, responses within cells may differ. Although our predictions provide some light on potential consequences, the true effects of SNPs in the HIC1 gene may be more intricate and diverse. To fully comprehend the range of SNP effects, experimental data from several biological contexts must be collected.

Limitations

In-silico analyses are theoretical and need experimental validation. The study was limited by the absence of laboratory trials to confirm the accuracy of the calculated predictions. The study utilizes existing databases for SNP information. Incomplete or erroneous data in databases may adversely affect the thoroughness and precision of the operation. If the SNP data used were biased toward certain ethnicities or groups, the results may not be applicable to other demographic groupings.

## Conclusions

To conclude, the use of in-silico analysis to analyze SNPs in the human *HIC1 *gene provided important insights into the possible functional impacts of genetic variants in this important signaling mechanism. We looked into the potential effects of SNPs on protein structure, function, and control using a variety of bioinformatics tools, databases, and prediction algorithms. This kind of in-silico research has identified a large number of SNPs that are projected to be hazardous, suggesting that they may be candidates for mutations linked to cancer that alter cells. The hic1 gene is impacted by many SNPs. Currently, research on cancer has demonstrated that SNPs can be used as a prognostic marker.
